# The association between caffeine intake and testosterone: NHANES 2013–2014

**DOI:** 10.1186/s12937-022-00783-z

**Published:** 2022-05-17

**Authors:** Frank E. Glover, William Michael Caudle, Francesco Del Giudice, Federico Belladelli, Evan Mulloy, Eniola Lawal, Michael L. Eisenberg

**Affiliations:** 1 Gangarosa Department of Environmental Health, Rollins School of Public Health, Emory University, Atlanta, GA 30322 USA; 2 Department of Maternal-Infant and Urological Sciences, “Sapienza”, Rome University, Policlinico Umberto I Hospital, Rome, Italy; 3 Department of Urology, Stanford University School of Medicine, Stanford, CA 94305 USA

## Abstract

**Background:**

Caffeine is one of the most commonly used psychoactive drugs in the world, and provides many health benefits including alertness, improved memory, and reducing inflammation. Despite these benefits, caffeine has been implicated in a number of adverse health outcomes possibly due to effects within the endocrine system, effects that may contribute to impaired reproductive function and low testosterone in men. Previous studies have investigated associations between caffeine consumption and testosterone levels in men, although the quantity and generalizability of these studies is lacking, and the results between studies are conflicting and inconclusive.

**Methods:**

Using data from a cross-sectional study of 372 adult men in the 2013–2014 NHANES survey cycle, the researchers set out to characterize the association between serum testosterone levels, caffeine, and 14 caffeine metabolites.

**Results:**

Multivariable, weighted linear regression revealed a significant inverse association between caffeine and testosterone. Multivariable, linear regression revealed significant, inverse associations between 6 xanthine metabolic products of caffeine and testosterone. Inverse associations were observed between 5-methyluric acid products and testosterone, as well as between 5-acetlyamino-6-amino-3-methyluracil and testosterone. A significant, positive association was observed for 7-methyl xanthine, 3,7-dimethyluric acid, and 7-methyluric acid. Logistic regression models to characterize the association between 2 biologically active metabolites of caffeine (theobromine and theophylline) and odds of low testosterone (< 300 ng/dL) were non-significant.

**Conclusions:**

These findings suggest a potential role for caffeine’s contribution to the etiology of low testosterone and biochemical androgen deficiency. Future studies are warranted to corroborate these findings and elucidate biological mechanisms underlying this association.

**Supplementary Information:**

The online version contains supplementary material available at 10.1186/s12937-022-00783-z.

## Introduction/Background

Caffeine is the most widely used psychoactive drugs in the world, and estimates show that over 80% of Americans consume caffeine daily, with an average consumption of more than 200 mg/day [1]. Coffee and tea represent the main sources of caffeine (> 80% of daily intake), while soda, energy drinks, and chocolate are minor sources [2, 3]. Analysis of caffeine and its metabolites has been of great interest with respect to population-level caffeine exposure, utility in kinetic and metabolism studies of CYP450 enzymes, and for use in in vivo studies to estimate caffeine’s association with health outcomes [4]. Numerous benefits of caffeine consumption have been documented, including improved mood and wakefulness, weight loss, antioxidative properties, and potentially improved long-term memory [5–7]. Despite these benefits, adverse symptoms of high caffeine consumption including restlessness, insomnia, dehydration, and cardiac abnormalities are well-documented [8]. Additionally, reports of adverse effects on several organ systems including the cardiovascular, neurological, and endocrine systems have been published [8–10]. In the context of the endocrine system, there is preliminary evidence to suggest caffeine and its metabolites exert effects on various pathways, including those related to testosterone biosynthesis [11, 12].

**Fig. 1:**
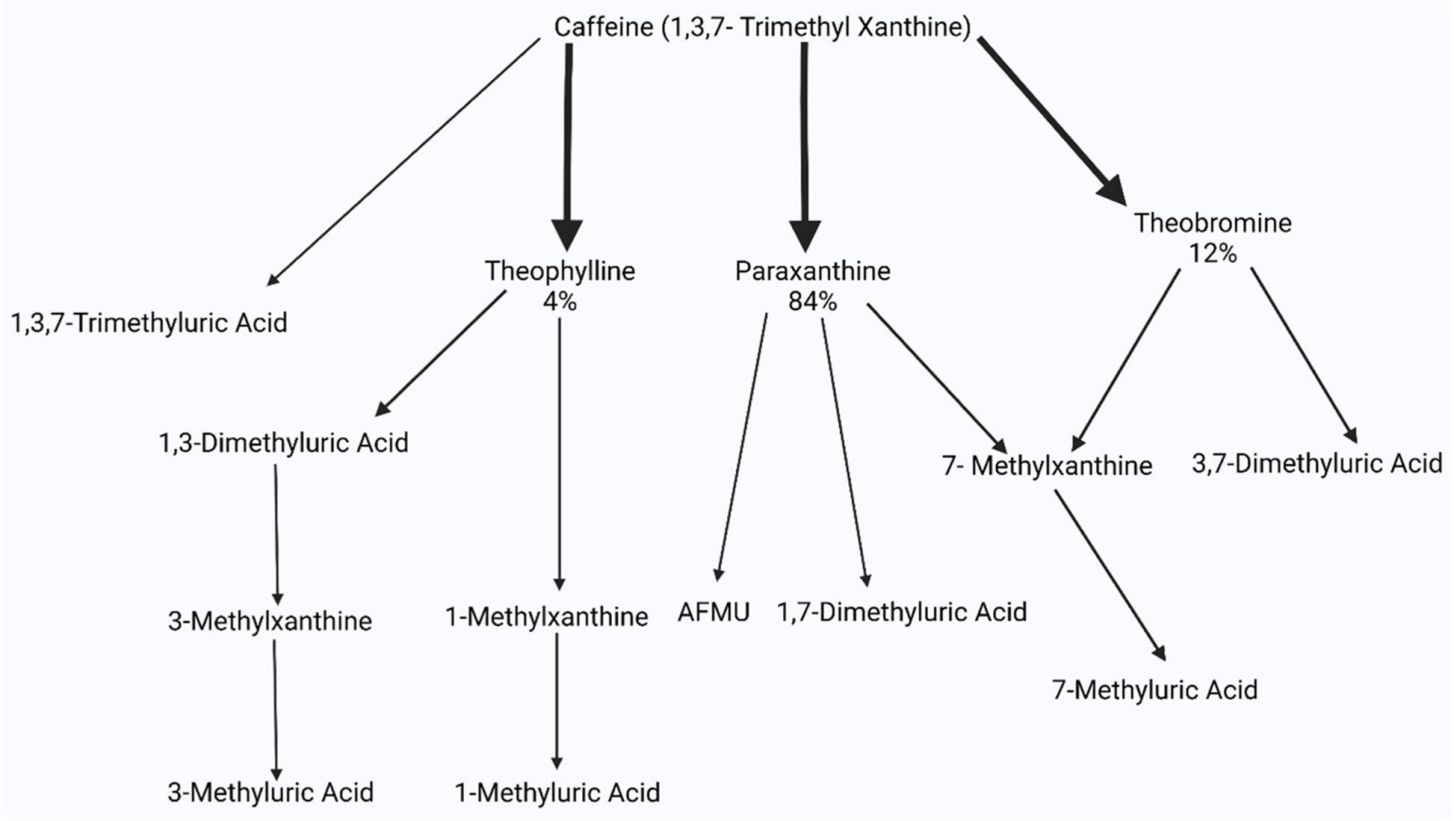
Showing the metabolism of caffeine into its various metabolites. Theophylline, Paraxanthine, and Theobromine represent the major metabolites of caffeine, denoted by the bold arrows (4%, 84%, and 12% respectively)

Caffeine has a mean elimination half-life of 5 h, and virtually all of caffeine is metabolized in the body, with only 3% or less being excreted unchanged in urine [13]. The cytochrome P450 enzymes in the liver, mainly CYP1A2, are responsible for the metabolism of caffeine, and population level differences in P450 enzymes are known to contribute to variations in caffeine metabolism [14]. The main route of metabolism in humans (70–80%) is through *N*-3-demethylation to paraxanthine, also known as 1,7-dimethylxanthine or 17X. 1-*N*-demethylation of caffeine to theobromine accounts for approximately 7 to 8% of caffeine metabolism, and 7-*N*-demethylation to theophylline also around 7 to 8% [15]. The remaining 15% of caffeine undergoes C-8 hydroxylation to form 1,3,7-trimethyluric acid (Fig. 1).

Testosterone plays an important role in men for the development of sexual features, brain function, muscle mass, and bone density [16, 17]. The most common symptoms associated with reduced serum testosterone (at or below 300 ng/dL) are decreased frequency of sexual thoughts and sexual desire, weight gain, and erectile dysfunction [18, 19]. In the U.S., the prevalence of low testosterone is as high as 40% in men over the age of 45 and is predicted to increase over the ensuing decades of life [20]. Paralleling the rise in low testosterone, there has been a decrease in fertilization rates both in the U.S. and worldwide [21]. Research shows that male factors such as hypogonadism account for over 40–50% of infertility cases [22–24]. A number of risk factors have been associated with low testosterone, including age, obesity, sedentary behavior, alcohol consumption, and certain medications [25, 26]. In addition to these risk factors, the potential role of the environment and dietary exposures, such as caffeine, on low testosterone etiology has come into light.

A number of population level, in vitro, and in vivo studies have investigated the effects of caffeine on testosterone levels, yet results of these studies remain conflicting, inconclusive, and lack generalizability to the U.S. adult population. In a study by Park et al., prepubertal male rats that were exposed to caffeine over four weeks showed significant reductions in serum testosterone, as well as reductions in testicular mass. In a study by Friedman et al., rats exposed to theobromine and caffeine experienced significant testicular atrophy, and plasma concentrations of testosterone were elevated in theobromine and caffeine-fed rats. In a study of human participants, Wedick et al. (2012) investigated the association between caffeinated and decaffeinated coffee on sex hormone binding globulin and endogenous sex hormone levels. Over the period of 8 weeks, no significant associations were observed, however at the 4-week interval, men showed an increase in total testosterone, while females showed a decrease in total testosterone. A previous NHANES study by Lopez et. al. found non-linear associations between recall caffeine intake and testosterone, but insignificant linear associations. Based on data from these previous studies, and the potential for caffeine and its metabolites to dysregulate testosterone pathways experimentally, the researchers hypothesized that caffeine consumption is significantly associated with testosterone in men. In a representative sample of U.S. adult men, the researchers set out to quantify the strength and direction of the association between caffeine and testosterone.

### Research design and methods

#### National Health and Nutrition Examination Survey (NHANES)

Data analyzed was collected from the 2013–2014 NHANES survey cycle (available from: https://wwwn.cdc.gov/Nchs/Nhanes/2013-2014/TST_H.htm). NHANES is a nationwide survey conducted annually for the purpose of collecting health and diet information from a representative, non-institutionalized U.S. population. NHANES is unique in that it combines interviews, physical examinations, and laboratory evaluations to obtain a large amount of quantitative and qualitative data. Information on NHANES survey methods are described in detail elsewhere [27]. Briefly, the survey examines about 5,000 persons each year from various counties across the U.S., which are divided into a total of 30 primary sampling units (PSUs), of which 15 are visited annually. All participants provided a written informed consent in agreement with the Public Health Service Act prior to any data collection. Household questionnaires, telephone interviews, and examinations conducted by healthcare professionals and trained personnel were utilized to collect data.

### Study participants and exclusion criteria

The 2013–2014 NHANES cycle collected data on 10,175 individuals. In the analysis, the researchers excluded a total 7,217 women and all children under the age of 18, leaving 2,958 men. Boys under 18 were excluded since low testosterone is a rare outcome in this age group and wouldn’t provide a sufficient sample size for robust analyses (n < 10). Those individuals presenting with low testosterone so early is likely attributable to genetic conditions or unusually high exposure to medications or toxicants, levels exceeding everyday exposure amounts [28, 29]. From these remaining individuals, analysis was restricted to men with valid serum testosterone concentrations, as well as complete information on demographic, anthropometric, questionnaire, and laboratory variables including BMI, alcohol use, diabetes status, creatinine and albumin concentration, ethnicity, smoking status, and sex hormone binding globulin concentrations, resulting in a final analysis sample size of 372.

### Assessment of serum testosterone

Following an overnight fast, serum samples were first taken between 8:30 a.m. and 11:30 a.m. and then testosterone concentrations were determined using a competitive electrochemiluminescence immunoassay on the 2010 Elecsys autoanalyzer (Roche Diagnostics, Indianapolis, IN, USA) with the lowest detection limit of the assay being 0.02 ng/mL. All sex steroid hormones from the present NHANES cycle were assayed at Boston Children’s Hospital (Boston, MA, USA) by laboratory technicians blinded to participant characteristics. The details for the NHANES laboratory methodology for testosterone determination are available from: https://wwwn.cdc.gov/Nchs/Nhanes/2013-2014/TST_H.htm.

### Quantification of caffeine metabolites

Caffeine and 14 of its metabolites were quantified in urine by use of high-performance liquid chromatography-electrospray ionization-tandem quadrupole mass spectrometry (HPLC–ESI–MS/MS), and with stable isotope labeled internal standards. With the exception of paraxanthine, which is readily obtained from plasma and minimally excreted in the urine, these metabolites are readily obtained from urine and serve as good proxies for caffeine exposure [30]. In addition to serving as proxies for caffeine, some of these metabolites are biologically active (e.g. methylxanthines, theophylline, theobromine), and may exert their own effects on endocrine pathways related to testosterone production. To begin sample preparation, 50-µL aliquots of urine were diluted with 450 µL of water. Then, 100 µL of the diluted urine was combined with 120 µL of a 0.2 N NaOH solution containing stable isotope labeled internal standards. The mixture was incubated for 30 min at room temperature. Samples were then acidified with 30 µL of 2.0 N HCl and 250 µL of a 1:9 methanol/water solution containing 0.1% formic acid. Quantitation by HPLC–ESI–MS/MS was based on peak area ratios interpolated against an 11-point calibration curve derived from calibrators in synthetic urine. A further detailed description on laboratory procedures can be found elsewhere [31].

### Defining demographic variables

Methods for questionnaire data collection are described in the NHANES procedures guide [32]. Covariates related to low testosterone, as well as potential confounders were included and based on results from literature searches. Participants were classified according to highest level of education attainment, insurance coverage status, smoking status, alcohol use, diabetes status, and cholesterol status. Levels of education were based on responses by participants during the home interview. Insurance coverage status and smoking status were recorded as a yes or no response from the home interview. Alcohol use was divided into three categories of “non-drinker”, “moderate drinker”, and “heavy drinker.” Non-drinkers were defined by individuals stating they drank less than 1 alcoholic beverage a week. Moderate drinkers reported drinking between 2–8 drinks a week. Heavy drinkers were defined as drinking over 10 alcoholic drinks a week. Diabetes status was defined as a fasting serum glucose greater than 126 mg/dL, having answered yes to taking diabetic medications, or having been diagnosed by a physician with diabetes. Cholesterol status was defined by whether or not a person was told he/she has high cholesterol by a physician, if the serum total cholesterol was greater than 240 mg/dL, and/or if that person is currently taking hypercholesterolemia medications.

### Statistical analyses

Continuous variables were compared using one-way ANOVA, while categorical variables were compared using the Chi-squared test. Multivariable, ordinary least squares regression models were used to measure the association between caffeine and its urinary metabolites, and serum testosterone concentrations. Additionally, theobromine and theophylline are biologically active metabolites with known involvement in pathways that may be related to testosterone production and maintenance. Therefore, theobromine and theophylline were included in the logistic regression models, to model the odds of low testosterone based on quartile of metabolite concentration. The lowest quartile was used as the reference in each case. The complex survey design assigns a weight to each individual as a function of their probability of being randomly selected and this was considered when building the regression models. The final models were adjusted for potential confounders including age, BMI, smoking, drinking, and creatinine to control for urinary dilution.

All statistical analyses were performed using SAS 9.4 and SUDAAN software packages accounting for the complex survey design of NHANES [33]. A *p*-value < 0.05 was used as the criterion for significance.

## Results

### Demographics of cohort

Table 1 shows the demographic breakdown of the cohort, and Table 2 shows the demographic breakdown of the cohort by quartile level of caffeine. With the exception of mean testosterone, mean sex hormone binding globulin, and mean caffeine concentration, no significant differences were found between quartiles of caffeine for the demographic and laboratory variables.

**Table 1:**
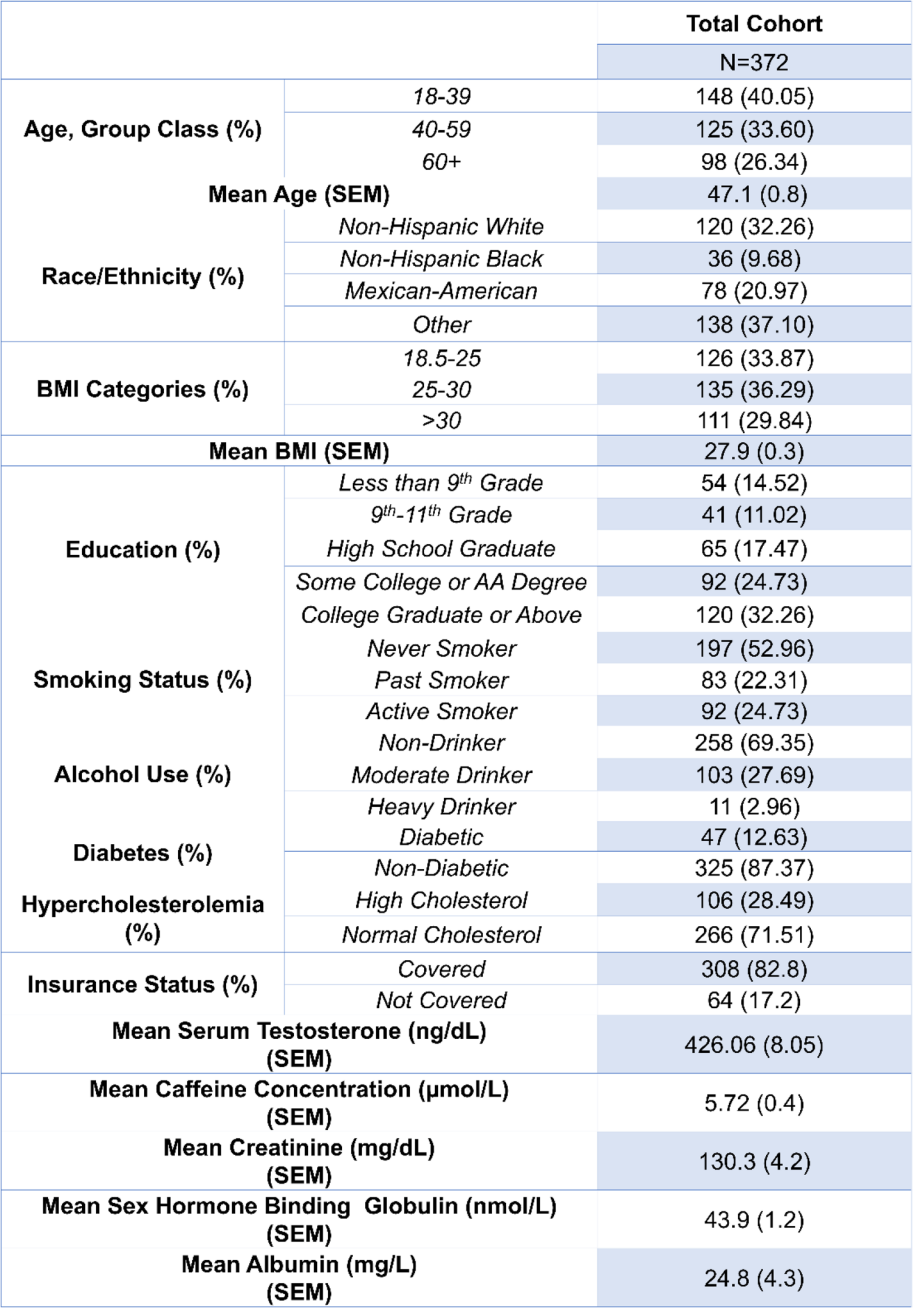
Showing the demographics data for the total cohort

**Table 2:**
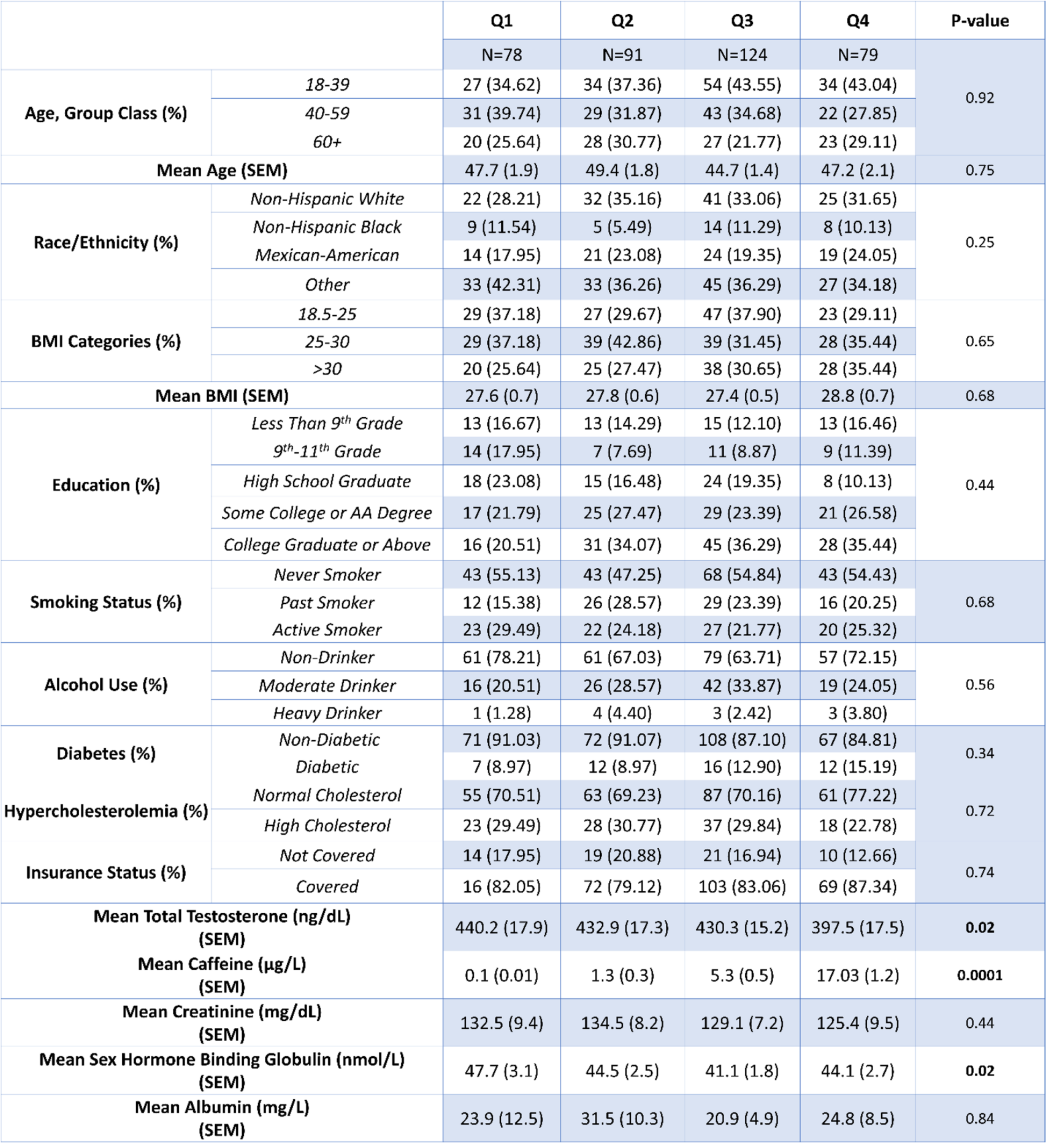
Showing the laboratory and demographic data by quartile of caffeine concentration. Comparisons of laboratory and demographic data between quartile levels were performed using one-way ANOVA for continuous variables, and chi-square for categorical variables. Statistical significance was set at p < 0.05

## Multivariable linear regression

The results from multivariable linear regression models are shown in Table 3. An inverse association between caffeine and testosterone was observed (β = -2.28 µmol/L, *p* < 0.0001). An inverse association between serum testosterone and 1-methyluric acid (β = -0.093215 µmol/L, *p* < 0.0001) was observed. A strong, inverse association was found between serum testosterone and 3-methyluric acid (β = -5.220847 µmol/L, *p* < 0.0001). A positive association was found between serum testosterone and 7-methyluric acid (β = 0.013838 µmol/L), though nonsignificant (*p* = 0.2262).

**Table 3:**
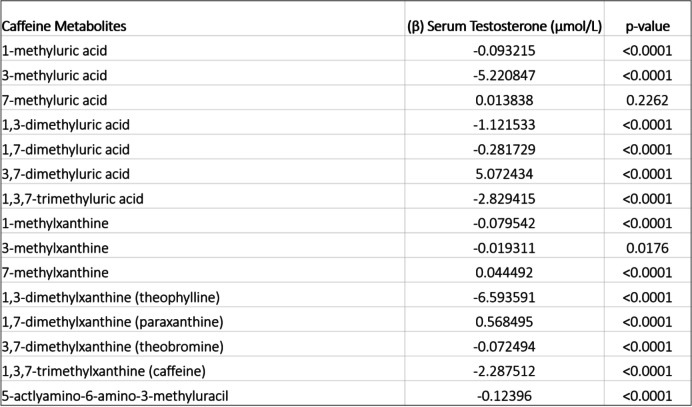
Showing the weighted, multivariable regression coefficients for caffeine and each metabolite. Statistical significance was set for *p* < 0.05

Metabolites 1,3-dimethyluric acid (β = -1.121533 µmol/L) and 3,7-dimethyluric acid (β = -0.281729 µmol/L) have an inverse association with serum testosterone (*p* < 0.0001). A strong, positive association was observed between serum testosterone and 3,7-dimethyluric acid (β = 5.072434 µmol/L, *p* < 0.0001). Inverse associations were observed for 1-methylxanthine and 3-methylxanthine (β = –0.079542 µmol/L, *p* < 0.001) and (β = –0.019311 µmol/L, *p* = 0.0176), respectively.

A positive association was observed between 7-methylxanthine (β = 0.044492 µmol/L, *p* < 0.0001) and testosterone. A strong, inverse association was found between testosterone and 1,3-dimethylxanthine and 1,3,7-dimethylxanthine (β = –6.593591 µmol/L, *p* < 0.0001) and (β = –2.287512 µmol/L, *p* < 0.0001), respectively. A regression coefficient value of –0.072494 µmol/L revealed a slightly inverse association for 3,7 dimethylxanthine and serum testosterone, with a statistically significant *p*-value < 0.0001. A positive association with testosterone was observed for 1,7 dimethylxanthine (β = 0.568494 µmol/L, *p* < 0.0001). Lastly, 5-actlyamino-6-amino-3-methyluracil revealed a negative association with serum testosterone (β = –0.12396 µmol/L, *p* < 0.001).

### Quartile and logistic regression

Subjects were stratified by quartile level of caffeine, theobromine, and theophylline exposure, and linear regression models were constructed to predict the mean change in testosterone level between each quartile. Logistic regression was performed to model the odds of having low testosterone for a given quartile of caffeine, theobromine, and theophylline. The lowest quartile was used as the reference in each case. Compared to the first quartile, those individuals in the second quartile of caffeine had an expected positive increase in mean testosterone (β = 59.11 ng/dL, *p* = 0.002), whereas those individuals in the fourth quartile compared to the first quartile had an expected decrease in mean testosterone (β = -28.84, p = 0.001). The second and third quartiles of theobromine showed a predicted decrease in mean testosterone compared to the first quartile (β = -74.95, *p* = 0.001, and β = -78.38, *p* = 0.01 respectively). The fourth quartile of theophylline showed a decrease in mean testosterone (β = -48.29 *p* = 0.01). Results from the logistic regression models were all non-significant. The results of these analyses are provided in supplementary tables.

## Discussion

There are several potential biological mechanisms underlying the associations observed between caffeine and testosterone. After ingestion, caffeine is known to exert various pharmacological effects at the cellular level [5]. Caffeine’s primary mechanism of action is antagonism of adenosine receptors, and acts on all four adenosine receptor subtypes in the brain (A1, A2a, A2b, A3) [34, 35]. In addition to those found in the brain, adenosine receptors have also been described in the testes [36]. Adenosine receptors observed in the testes are mainly localized within the Leydig and Sertoli cells of the seminiferous tubules, and these receptors are associated with an inhibition of cellular responses following activation. Following activation of these receptors, cAMP/protein kinase pathways, which are usually activated in mediation of testosterone production, are downregulated and may lead to lower testosterone production [37–39]. It is possible that caffeine affects testosterone production through these adenosine-dependent pathways.

The findings of this study may also be relevant for early life exposure to caffeine, and long-term effects exposure may have on reproductive outcomes. Reproductive studies have begun to investigate the association between caffeine exposure and parameters of reproduction such as sperm quality, semen volume, and egg maturation. A previous by Dlugosz et. al found that doses of caffeine higher than 400 mg/day might decrease sperm motility and/or increase the percentage of dead spermatozoa, but not sufficiently to affect the male fertility in an adverse manner [40].Rats exposed to high doses of caffeine in utero developed smaller testes compared to controls [41]. This study also found that stimulated-testosterone ex vivo production was reduced in Leydig cells retrieved from the high-dose caffeine rats. A Danish pregnancy cohort study found that men who were born to mothers drinking 4–7 cups/day of coffee had lower testosterone levels than sons of mothers drinking 0–3 cups/day [42, 43]. Furthermore, there was a significant, positive association between high caffeine intake and testosterone levels in the adult males. In addition to direct effects on the testes, caffeine has been shown in vitro to induce aberrant DNA methylation and histone acetylation of the steroidogenic factor-1 (SF-1) promoter in the rat fetal adrenals, which acts to reduce transcription of the SF-1 gene. SF-1 is a key transcription factor involved in transcription of genes related to steroidogenesis and testosterone biosynthesis in males, and reduced expression of SF-1 may be a mechanism of low testosterone related to caffeine.

In addition to caffeine, the metabolically active products of caffeine metabolism including theophylline and theobromine of the xanthine class have been shown to have direct effects on gonadotropin-induced steroidogenesis [44]. Mechanistically, theophylline and theobromine act as a phosphodiesterase inhibitors, adenosine receptor blockers, and histone deacetylase activators [45, 46]. In one study, various concentrations of theophylline and 1-methyl 3-isobutyl xanthine (MIX) significantly inhibited steroidogenesis. Additionally, higher concentrations of MIX and theophylline also significantly inhibited precursor incorporation into RNA and protein. In another study, Osborne-Mendel rats who were fed varying doses of caffeine, theophylline, and theobromine exhibited significant testicular atrophy and impaired steroidogenesis [47].Thus, in addition to direct effects of the parent compound caffeine, caffeine’s biologically active metabolites may also act through various pathways to affect testosterone production and half-life.

The present study has a number of strengths. By using the NHANES census data, the researchers were able to incorporate a large number of men representative of the general U.S. adult population, and able to characterize the association between testosterone and daily exposure levels of caffeine. Previous studies have limited their analyses to indirect quantitative associations between caffeine consumption and testosterone. For example, prior to this NHANES survey cycle, recall data on number of cups of coffee per day has generally been used as a surrogate for caffeine consumption. A previous NHANES study by Lopez et. al analyzed 24-h dietary recall data to estimate caffeine intake, and investigate the association between caffeine and testosterone [48]. They found no linear associations between caffeine intake and testosterone, but did observe non-linear associations between caffeine intake and testosterone. An improvement on this study is the measurement of caffeine and its metabolites directly. Additionally, by collecting data on specific metabolites of caffeine the researchers are able to characterize potential direct associations between biologically active metabolites of caffeine (e.g. theophylline and theobromine) and testosterone levels. As this study reveals differential associations between testosterone levels among the metabolites, it is possible that different classes of caffeine metabolites exert similar or unique effects on the biochemical processes related to testosterone production. Future studies are warranted that test these individual effects of these metabolites on various biochemical pathways affecting testosterone.

Due to the cross-sectional nature of the study, this study cannot draw any true cause and effect conclusions between caffeine, its major metabolites, and testosterone levels. Additionally, the researchers are unable to determine the specific source of caffeine in this cohort (e.g. coffee, tea, soda etc.). Different sources of caffeine such as soda vs. coffee can present their own unique health profiles, which could affect organ systems that regulate testosterone production differentially [49]. Furthermore, special subpopulations of the U.S. may be more susceptible to potentially adverse effects of caffeine. One example would be individuals who have varying levels of endogenous cytochrome P450 enzymes, the enzyme class responsible for caffeine metabolism. Numerous medications and even some foods (i.e. grapefruit juice) are known inducers or inhibitors of cytochrome P540 enzymes. Additionally, studies have shown that naturally occurring polymorphisms in P450 enzymes directly affect the half-life of caffeine, which in turn affects the exposure duration of patients to caffeine and its health effects [50–52]. Lastly, using cross-sectional data this study unable to determine potential dose–response effects of caffeine on testosterone. As previously mentioned, studies have shown that for low dose of caffeine exposure, the effects on testosterone levels are positive, and for high caffeine levels inverse associations between caffeine and testosterone were observed. Future studies that include delineating sources of caffeine, include various special populations, evaluate dose–response effects, and track early caffeine exposure are warranted.

## Conclusion

In a nationally representative sample of U.S. adult men, the researchers observed an inverse association between caffeine and serum testosterone. Given the popularity of caffeine consumption, it is both a clinical and public health priority to understand the biological effects of caffeine on testosterone levels and associated endocrine pathways. These effects of caffeine may serve as important risk factors in the etiology of low testosterone and reproductive dysfunction. Future studies are warranted to corroborate these findings, and to investigate biological mechanisms potentially responsible for caffeine’s effect on testosterone.

## Supplementary Information

**Additional  file 1.** Supplementary Tables 1-3

## Data Availability

A full list of data sets supporting the results in this research article can be found at: https://wwwn.cdc.gov/nchs/nhanes/continuousnhanes/default.aspx?BeginYear=2013.
