# Supplementary material for: The association between caffeine intake and testosterone: NHANES 2013–2014
Source: Nutr J. 2022 May 17;21:33. doi: 10.1186/s12937-022-00783-z (PMC9112543; doi:10.1186/s12937-022-00783-z)
Supplement: Supplementary file 1 — Additional file 1. Supplementary Tables 1-3 [file 12937_2022_783_MOESM1_ESM.docx]

Supplementary Table 1


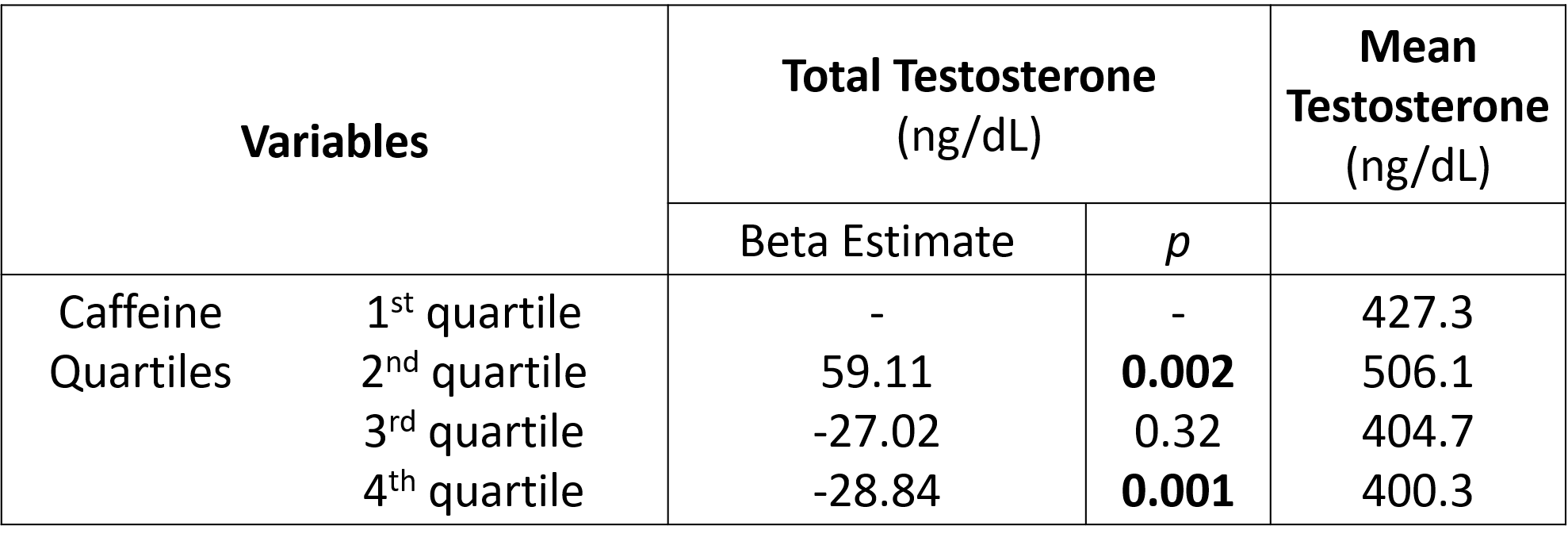


Supplementary table showing the beta estimates for expected change in testosterone for each quartile of urinary caffeine concentration. The first quartile was used as the reference in each case. A p-value <0.05 was used as the cutoff for statistical significance.

Supplementary Table 2


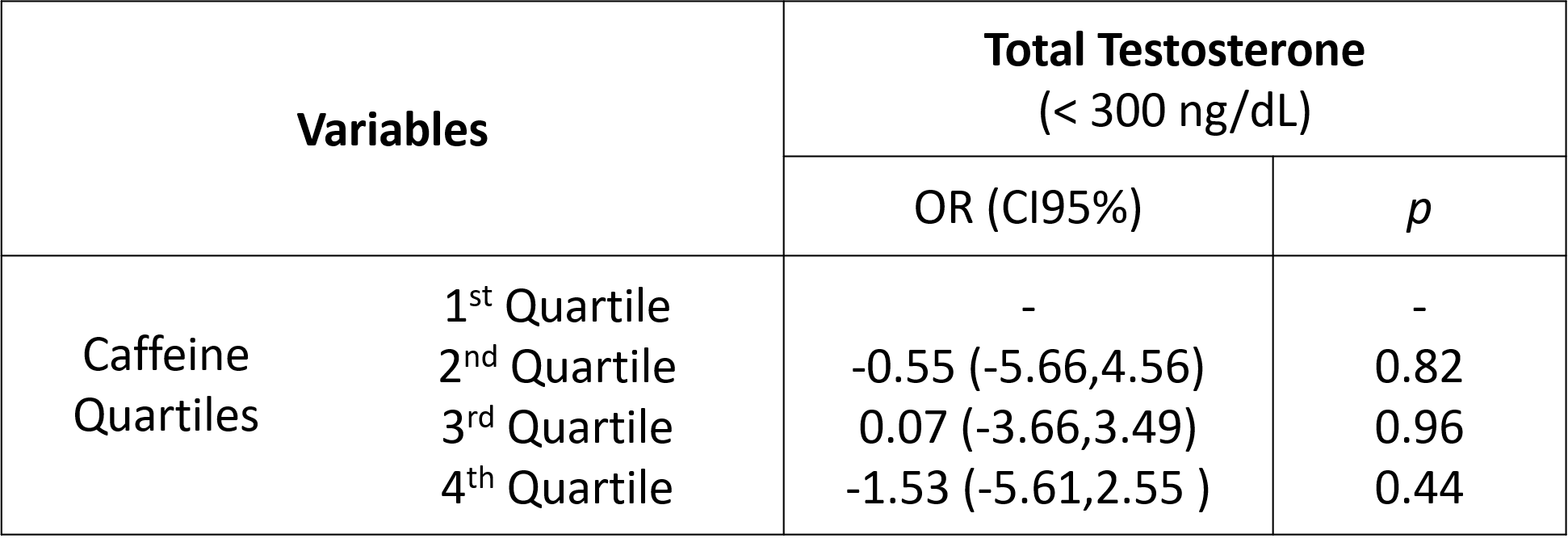


Supplementary table showing the odds ratios for low testosterone given each quartile of caffeine. Quartile 1 was used as the reference in each case. A p-value of <0.05 was used as the cutoff for statistical significance.

Supplementary Table 3


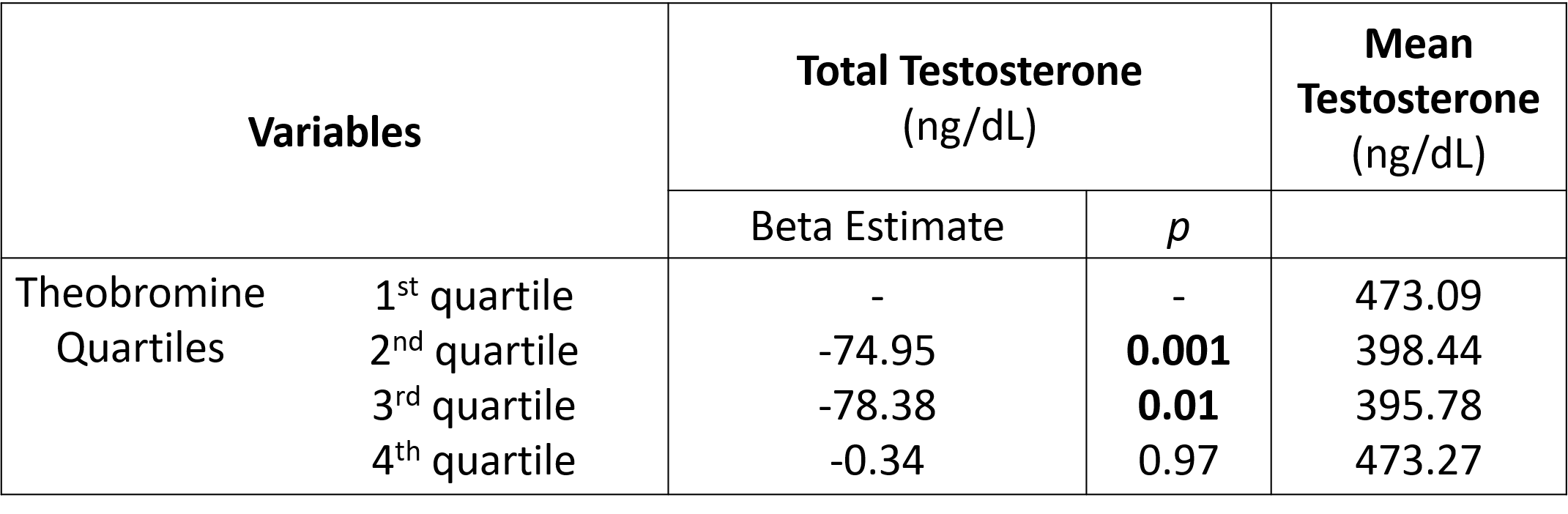


Supplementary table showing the beta estimates for expected change in testosterone for each quartile of urinary theobromine concentration. The first quartile was used as the reference in each case. A p-value <0.05 was used as the cutoff for statistical significance.

Supplementary Table 4


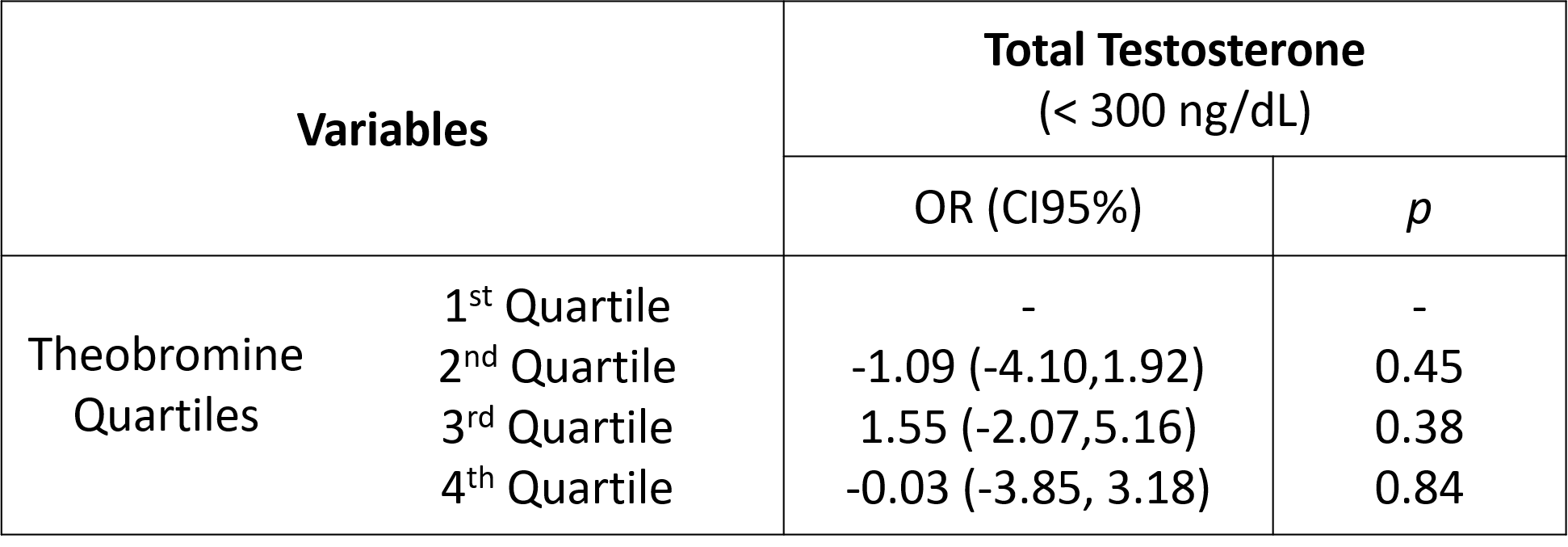


Supplementary table showing the odds ratios for low testosterone given each quartile of theobromine. Quartile 1 was used as the reference in each case. A p-value of <0.05 was used as the cutoff for statistical significance.

Supplementary Table 5


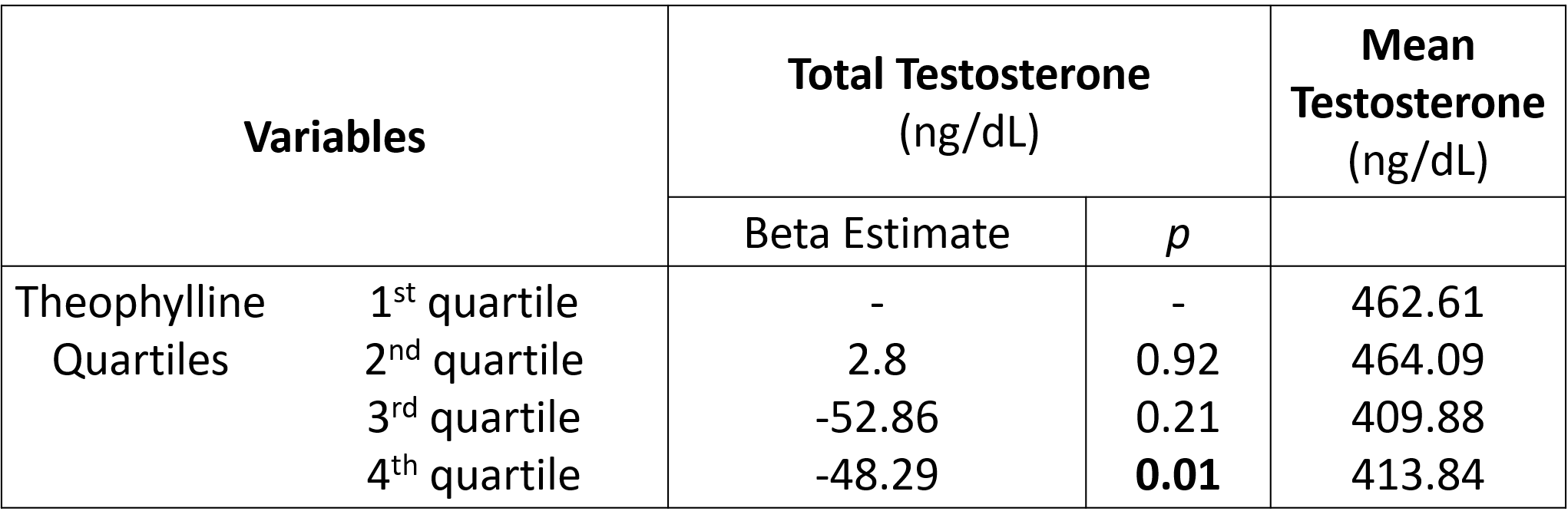


Supplementary table showing the beta estimates for expected change in testosterone for each quartile of urinary theophylline concentration. The first quartile was used as the reference in each case. A p-value <0.05 was used as the cutoff for statistical significance.

Supplementary Table 6


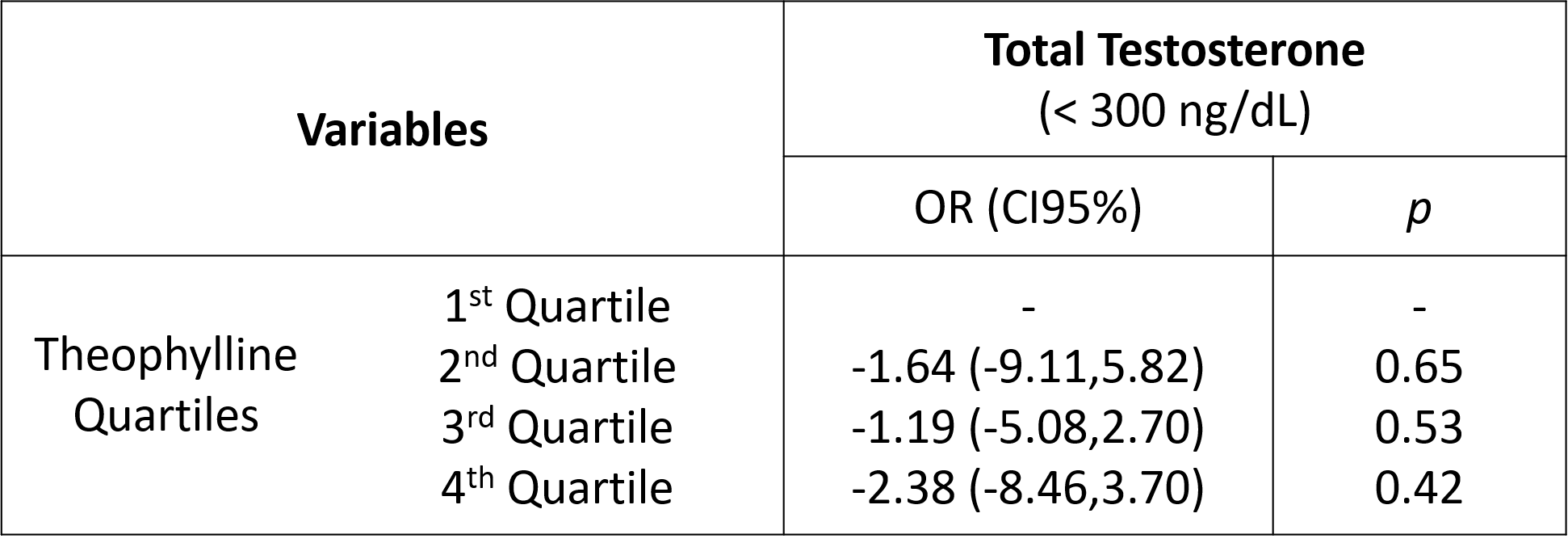


Supplementary table showing the odds ratios for low testosterone given each quartile of theophylline. Quartile 1 was used as the reference in each case. A p-value of <0.05 was used as the cutoff for statistical significance.
